# Muscles adaptation to aging and training: architectural changes – a randomised trial

**DOI:** 10.1186/s12877-020-02000-0

**Published:** 2021-01-13

**Authors:** Adrien J. Létocart, Franck Mabesoone, Fabrice Charleux, Christian Couppé, René B. Svensson, Frédéric Marin, S. Peter Magnusson, Jean-François Grosset

**Affiliations:** 1 Sorbonne Universités, Biomécanique et Bioingénierie, Université de Technologie de Compiègne, UMR CNRS 7338, Compiègne, France; 2 Centre Hospitalier COMPIÈGNE-NOYON, Compiègne, France; 3 ACRIM-Polyclinique Saint Côme, Compiègne, France; 4 Institute of Sports Medicine Copenhagen / Dept of Physical Therapy, Bispebjerg Hospital, Copenhagen, Denmark; 5 Center for Healthy Aging, Faculty of Health and Medical Sciences, University of Copenhagen, Copenhagen, Denmark

**Keywords:** Muscle volume, Resistance training, Ageing, Anatomical cross-sectional area, Non-contractile tissue

## Abstract

**Background:**

To investigate how anatomical cross-sectional area and volume of quadriceps and triceps surae muscles were affected by ageing, and by resistance training in older and younger men, in vivo.

**Methods:**

The old participants were randomly assigned to moderate (O55, *n* = 13) or high-load (O80, *n* = 14) resistance training intervention (12 weeks; 3 times/week) corresponding to 55% or 80% of one repetition maximum, respectively. Young men (Y55, *n* = 11) were assigned to the moderate-intensity strengthening exercise program. Each group received the exact same training volume on triceps surae and quadriceps group (Reps x Sets x Intensity). The fitting polynomial regression equations for each of anatomical cross-sectional area-muscle length curves were used to calculate muscle volume (contractile content) before and after 12 weeks using magnetic resonance imaging scans.

**Results:**

Only Rectus femoris and medial gastrocnemius muscle showed a higher relative anatomical cross-sectional area in the young than the elderly on the proximal end. The old group displayed a higher absolute volume of non-contractile material than young men in triceps surae (+ 96%). After training, Y55, O55 and O80 showed an increase in total quadriceps (+ 4.3%; + 6.7%; 4.2% respectively) and triceps surae (+ 2.8%; + 7.5%; 4.3% respectively) volume. O55 demonstrated a greater increase on average gains compared to Y55, while no difference between O55 and O80 was observed.

**Conclusions:**

Muscle loss with aging is region-specific for some muscles and uniform for others. Equivalent strength training volume at moderate or high intensities increased muscle volume with no differences in muscle volume gains for old men. These data suggest that physical exercise at moderate intensity (55 to 60% of one repetition maximum) can reverse the aging related loss of muscle mass.

**Trial registration:**

NCT03079180 in ClinicalTrials.gov. Registration date: March 14, 2017.

## Background

Muscle ageing is known to be associated with a marked decrease in muscle mass and physical function. It is estimated that this age associated atrophy reduces lean muscle mass by 1–2%/year beyond 50 years of age depending on the muscle group and the person’s level of activity [1]. This decrease in mass is more pronounced for the lower limbs than for the upper limbs, and it has been estimated that between 20 and 70 years the muscle mass of the lower limb is reduced by ~ 25% [2]. A decrease in strength and power has been reported in a senior male population compared to young subjects [3, 4], particularly in the quadriceps and triceps surae (TS) muscles. It has been reported that quadriceps muscle volume was lower in old men than young men with a significant decline in mid-thigh area, but no value was reported in other part of quadriceps muscle [5]. A better understanding of muscle atrophy is necessary to effectively prevent or minimize this phenomenon. However, it remains unknown if the muscle loss is a homogeneous phenomenon or if there is a region-specific muscle loss with age.

Physical exercise, and particularly resistance training, could prevent and combat sarcopenia [6, 7]. However, the intensity required to maximize muscle strength gains and/or sarcopenia reduction remains unclear despite several reviews (training exercise with intensities between 50 to 80% of one Repetition Maximum (1RM)) [8, 9]. It has been shown that the rate of muscle protein synthesis reaches a maximum at 60% of 1RM in the elderly, unlike younger subjects who see increased synthesis all the way up to 90% of 1RM [10]. Therefore, one could hypothesize that training at intensity greater than 60% of 1RM would not lead to additional gains in muscle mass in the elderly.

An accurate measurement of muscle volume is a prerequisite to investigate how muscle mass contributes to changes in muscle force with aging [11], training [7, 12, 13], growth [14], gender [15], immobilisation [16] or disease [17, 18]. However, muscle hypertrophy/atrophy is often investigated based on a single cross-sectional area (CSA) measurement for time and cost reasons, and this may not adequately reflect changes in muscle volume [7].

To accurately estimate the entire muscle volume, the use of continuous transverse magnetic resonance imaging (MRI) scans has been validated [19] and widely used [12, 13, 20, 21]. Assessment of muscle volume could be calculated using area under the fitting curve obtained from CSA analysis over the entire muscle length, or estimated from a single scan using a curve previously published [22], or using the truncated cone method [23]. Nevertheless, the curves established by Morse et al. [22] were established only for quadriceps muscles, extrapolated for a portion, and on a population of young people, so it is unknown if they can be applied to older persons and if the same holds for triceps surae muscle (TS).

In view of the above, we therefore investigated the effect of aging and physical activity with the aim of 1) establishing specific reference curves of ACSA (anatomical cross-sectional area) in young and old men for the quadriceps and triceps surae muscles; 2) relating the shape (ACSA evolution) of these muscles with age; 3) comparing pre and post training curves in young and old males at moderate and high training intensities; 4) making a comparison finally with the effect of a defined training volume on different muscles groups (quadriceps vs. TS). We hypothesized that aging displays a homogeneous phenomenon on ACSA evolution on muscle. The second hypothesis was that the elderly have same benefit due to training from different exercise intensities (identical volume gain between moderate and high training loads).

## Methods

### Participants

Eleven recreationally active young men and 35 sedentary elderly men were recruited to participate in this study. The present study is a part of a larger study, designed to evaluate the effects of training intensity and age on changes in tendon architecture and its mechanical properties, lower limb muscle adaptations, and motion capacity. This sample size was determined by a preliminary power analysis (Continuous outcome superiority trial, α = 0.05, 1-β = 0.90) and performed with Sealed Envelope software (Sealed Envelope Ltd. 2012, London, UK), based on previously published data showing significant increase in cross-sectional area (CSA) of the muscular and tendon systems by MRI using a similar training protocol [7, 12, 24–28]. With these parameters the required population size had to be composed by 12 subjects in each group. Their physical activity level was primarily determined through their leisure time activities assessed by a PASE questionnaire [29]. Only participants with a PASE score < 150 were included. Each included participant completed a medical questionnaire and was examined by an orthopedic doctor. Briefly, all participants were healthy normotensive (< 140/90 mmHg) and non-obese or anorexia (20 < BMI < 28), did not take any prescription medication, had no overt symptoms of diabetes, atherosclerosis, or any joint, muscle or tendon pathology, and without metallic implants/objects. All of the participants were fully informed regarding the experimental procedure and gave their written informed consent. During the training intervention, participants were recommended to abstain from other physical activities. The experimental protocol was approved by the local ethical and personal protection committee (CPP-2016/52) and registered on ClinicalTrials.gov (NCT03079180). The participants were free to withdraw from the study at any time. Our study adheres to CONSORT guidelines [30].

### Study design

The size of lower limb muscles was determined from multiple MRI scans before and after a 12-week resistance training program as described in detail below. To prevent an influence of fluid shifts on muscle volume, the MRI scans were performed at the same time each day [31]. Two pre-test scans were performed (4 weeks and just before the beginning of training) and the first scan was used as a ‘control’. Post-test measurements were performed 4 days after the last training session. Values of each parameter obtained during the pre-training sessions were used to investigate the effect of aging.

### Resistance training intervention

The young participants were assigned to a moderate-load training intervention corresponding to 55% of 1RM (Y55). The old participants were randomly assigned to either moderate (O55) or high-load (O80) training intervention corresponding to 55% or 80% of 1RM, respectively. The training protocols were designed to produce similar total volumes of work in both load training groups. Participants took part in a 12-week training program (three times a week) for the muscle groups of the triceps surae and quadriceps using seated calf extension, leg extension and seated leg press (Life-Fitness, Rosemont, Illinois, USA). All training sessions were supervised by a member of the research team in order to correctly teach the exercises to be performed, to check on each series that they were performed correctly, and if necessary to apply corrections ensuring that the exercises were always performed in total safety for the participants. For each participant, the determination of the maximum load (1RM) that could be developed was carried out at the first session, readjusted every 15 days and performed prior to the normal exercise session. The determination of the maximum load for each exercise was performed as follows: a standard warm up was performed consisting of six repetitions at 50% of the maximum perceived force (using a 10-point Borg Resistance Exercise Scale of Perceived Exertion [32] and four repetitions at 70% with 3 min rest between the two series. Subsequently, a series of single lifts were performed with 3-min rest intervals at increments of 5 kg if the preceding lift was successful (full knee extension or plantar flexion). The weight at the last successful lift was defined as 1RM. Each training session (duration ~ 1 h) consisted of a warmup of 10–15 min on a cycle ergometer (Technogym Bike Excite, Gambettola, Italy). Then a defined number of repetitions (details in Table 1) (3 s in concentric, 3 s in eccentric) at 55 or 80% 1RM depending on the assignment group, and a specified number of series on each device were performed. The total training volume for the groups was similar (number of repetitions x number of series x relative-load (corresponding at % of load) and matched for both muscle groups (double series on the calf machine compared to the other two devices that use the knee extensor muscles (Leg-extension and leg press)). In addition, to reduce risk of injury at the start of training, the high intensity senior group had a progressive program with increasing load during the first 6 weeks of training to reach the target training intensity of 80% of 1RM from the seventh week of training (Table 1). Although the investigations focused on the muscles of the right leg, the training sessions were applied to both legs in order to induce harmonious development and thus avoid causing an imbalance between the two legs.

**Table 1:** Distribution of training volume according to training intensity

	Week								
		1	2	3	4	5	6	7–12	
**High intensity** **(80% 1RM)**	Repetition	15	12	10	8	6	5	4	
	Intensity	55	55	55	65	70	75	80	
	Series	3	3	3	4	4	4	5	
									Total volume
	Week volume	2475	1980	1650	2080	1680	1500	1600	12,965
**Moderate intensity** **(55% 1RM)**	Repetition	15	12	10	9	8	7	6	
	Intensity	55	55	55	55	55	55	55	
	Series	3	3	3	4	4	4	5	
									Total volume
	Week volume	2475	1980	1650	1980	1760	1540	1650	13,035

### Experimental outcomes

#### Muscle dimensions

MRI scans were acquired along the length of the right tibia (for triceps surae muscles) and femur (for quadriceps muscles) using a magnetic resonance imaging (MRI; 1.5 T Signa HDx, General Electric, Milwaukee, WI, USA). Participants laid on the MRI table on their back. The ankle was held at 90° by a custom wooden square to ensure the same position between participants and test sessions. A specific antenna for the acquisition of muscle cross-sectional areas (CSA) was installed (covering 45 cm). The femoral quadriceps and triceps surae muscles were scanned using a VIBE (Volume interpolated GRE) image sequence (Matrix 240 × 240; TR 6.3 ms; TE 3.0 ms, cutting thickness 2.6 mm, gap = 0). An axial scan was performed perpendicular to the thigh, from the femoro-tibial joint to the iliac crest for the quadriceps muscles, and from the calcaneus to the femoro-tibial joint for triceps surae muscles. Due to the length of the antenna, the quadriceps scan had to be performed with two sequences for some tall participants (*n* = 14). In this case, external markers (oil capsules) were placed on the participant’s leg using adhesive tissue to be able to adjust and overlap the different acquisition sequences in post treatment.

#### Data processing

The accuracy of using serial ACSA scans from MRI for the measurement of muscle volume has been reported previously [19, 33]. The anatomical cross-sectional area (ACSA) was measured by an image processing software (Simpleware ScanIP, Synopsys Inc., Exeter, United Kingdom) throughout the excursion of each of the individual muscles composing the two muscle groups of interest: triceps surae and quadriceps. The distal and proximal insertions of each of the muscles analyzed were identified before starting the analysis. For each of the seven individual muscles of interest (quadriceps muscles (VL, VI, VM, RF) and triceps surae muscles (GL. GM, soleus)), segmentation was performed every eight slices (18.2 mm). In the first of this analysis, the ACSA of each muscle was tracked manually and measured digitally, and this was done for the three test sessions of a same participant by the same investigator. Furthermore, a grayscale thresholding of the MRI image was used to identify the aponeurotic limit. In a second step, visible fat and connective tissue were excluded from the measurement to only take into account the contractile part of the muscle volume [34]. To separate the non-contractile tissue, a region of interest (ROI) containing muscle and subcutaneous fat was made. Then, a histogram of signal intensity within the ROI was produced. To separate contractile and non-contractile tissues with minimal investigator bias, thresholding was performed by the Maximum Entropy method, a reliable histogram shape-based technique used in medical imaging analysis [35]. Pixel values above the threshold were considered to be non-contractile tissue [18]. Finally, this area, comprising fat and connective tissue, was then subtracted from the total muscle area, in order to measure only the CSA of the contractile components.

#### Regression equations

To be able to compare the ACSA of each participant according to their muscle length, the position of the ACSA was expressed according to the relative position of the ACSA in relation to the total length (L) of the muscle of interest. The position corresponding to 0% muscle length represented distal insertion for the quadriceps muscles, and proximal insertion for the triceps surae muscles with 100% representing the proximal and distal insertions for the quadriceps and triceps surae muscles respectively [22, 36]. To compare the evolution profiles of the ACSA with age and training, the ACSA values obtained were normalized to the maximum ACSA (ACSA_max_, cm^2^) value reached for each of the muscles analyzed. The evolution of the ACSA of each of the seven muscles of interest was then expressed as a function of the relative length of the muscle and a polynomial regression curve was fitted to the data.

#### Estimated muscle volume

The volume of each muscle was estimated by integrating the regression equation (function of ACSA_max_) over the length of the muscle (L) using the formula:

$$ \mathrm{V}={\int}_0^1f{(ACSAmax)}_x\times {length}_{muscle}\  dx $$

The length of the muscle (L, cm) is the distance between the proximal and distal insertion of the considered muscle (corresponding to the number of MRI slices x slice thickness (2.6 mm)) [22].

#### Statistics

The statistical analyses were performed with SigmaPlot 11.0 software (Systat Software, San Jose, USA).

Root mean square (RMS) differences were used to assess reproducibility from the two pre-training data sets (4 weeks and just before the beginning of training) on regression curves. Intraclass correlation (ICC) was calculated for muscle volume comparison from both measurement sets.

Two-way analysis of variance (ANOVA) (Holm–Sidak multiple comparisons post hoc testing) with distance (every 25% of the relative length of the muscle) and age (between group young vs. old) factors, was used to determine differences on the mean value of ACSA in the atrophy in individual muscles. Similarly, the effect of training on the mean value of ACSA within group (pre vs. post) every 25% of the relative length of the muscle was analyzed using the same two-way ANOVA.

After testing the normality distribution using a Shapiro-Wilk test, the comparison of the volume between pre and post-test, was tested by a Paired t-test. Values were considered significant at an alpha level of *P* < 0.05. The effect of training between the three different groups and between quadriceps and TS muscles within the same group (Y55, O55, O80) were analyzed using two-way ANOVA, with Holm–Sidak multiple comparisons post hoc testing. Data are presented as means ± SD.

## Results

### Participants

Of a total of 46 male participants initially included in the study, 27 elderly and 10 young male participants completed the protocol. Their characteristics are presented in Table 2. Over the nine participants who did not complete the entire experimental protocol, one had to be excluded for non-compliance with the training protocol, four were unfortunately affected by a pathology requiring treatment incompatible with our protocol, and four contracted an injury outside training. Concerning the analysis of the participant characteristics of the different groups, as expected there is a significant difference in average age between the youth group and the senior groups (*p* < 0.001). For all other parameters (height and weight), no significant differences were observed between the groups (*p* > 0.05).

**Table 2:** Participants characteristics

Variable	Young trained at 55% 1RM (Y55)	Old trained at 55% 1RM (O55)	Old trained at 80% 1RM (O80)
Number of participants	**10**	**13**	**14**
Age (Years)	24.8 ± 3.6*	**70.0 ± 4.6**	**69.8 ± 4.4**
Height (cm)	**178.8 ± 7.5**	**175.0 ± 7.2**	**176.3 ± 4.8**
Weight (Kg)	**75.2 ± 11.6**	**80.3 ± 12.4**	**80.3 ± 13.6**

*MVC* Maximal Voluntary Contraction

*Significant difference between young and old (S55 and S80) (*p* < 0.001); Data are presented as means ± SD

### Reliability

For all the investigated parameters, ICC and RMS differences in muscle volume of the 7 investigated muscles before training were calculated as presented in Table 3. The results indicate an RMS difference of the CSA below 1.0 ± 0.5 cm^2^ for all the curves of the different muscles. The ICC values ranged from 0.79 ± 0.04 to 0.90 ± 0.01 for each muscle volumes.

**Table 3:** ICC values and RMS differences of each investigated muscle

Muscle	ICC for muscle volumes	RMS differences for muscle curves (cm^2^)
VL	0.83 ± 0.02	0.7 ± 0.2
VI	0.80 ± 0.04	0.4 ± 0.1
VM	0.79 ± 0.03	0.6 ± 0.2
RF	0.90 ± 0.01	0.6 ± 0.1
MG	0.82 ± 0.05	0.8 ± 0.3
LG	0.85 ± 0.03	0.7 ± 0.2
Sol	0.79 ± 0.04	1.0 ± 0.5

Vastus Lateralis (VL), Vastus Intermedius (VI), Vastus Medialis (VM), Rectus Femoris (RF) Medial gastrocnemius (MG), Lateral gastrocnemius (LG), Soleus (Sol). ICC: intraclass correlation. RMS: root mean square differences. Data are presented as means ± SD

For age and training effects, the mean values of each parameter obtained during the two pre-training sessions (4 weeks and just before the beginning of training) were not significantly different (*p* > 0.6), therefore the values obtained in these two tests were averaged to get the pre-training value.

### Regression model

In order to better correspond to the anatomy we chose the smallest degree of the polynomial adjustment curve that passed through proximal and distal origins of the muscle and had a sufficient determination coefficient (R^2^ > 0.8).

By comparing different polynomials of different degrees, a 3rd degree polynomial adjustment for quadriceps muscles (VL, VI, VM, RF), and a 4th degree polynomial for triceps surae muscles (GL. GM, soleus) were used. All regression constants for each muscle for the young and old groups are shown in Table 4.

**Table 4:** Regression constants for each muscle for young and old group

Muscles	a	b	c	d	R^2^
**Young Group**					
VL	**− 2.6917**	**0.2544**	**2.4614**	**/**	**0.9661**
VI	**−1.5859**	**−1.4222**	**2.9428**	**/**	**0.9594**
VM	**6.1739**	**−12.432**	**6.3191**	**/**	**0.951**
RF	**−4.5326**	**3.0291**	**1.4916**	**/**	**0.9757**
MG	**3.1543**	**−5.3238**	**−1.563**	**3.6899**	**0.9457**
LG	**13.251**	**−25.583**	**11.267**	**1.0653**	**0.9661**
Sol	**9.2596**	**−14.119**	**1.4893**	**3.4211**	**0.9466**
**Old group**	a	b	c	d	R^2^
VL	**−2.4881**	**−0.0495**	**2.5442**	**/**	**0.9634**
VI	**−1.4025**	**−1.7304**	**3.0576**	**/**	**0.9375**
VM	**6.3957**	**−12.697**	**6.3731**	**/**	**0.9451**
RF	**−3.9678**	**2.2447**	**1.6946**	**/**	**0.9577**
MG	**7.9128**	**−16.14**	**6.3253**	**1.8729**	**0.9727**
LG	**10.614**	**−20.573**	**8.3903**	**1.5649**	**0.9584**
Sol	**11.127**	**−18.016**	**4.0566**	**2.8921**	**0.9465**

Constants a,b,c refers to for 3rd polynomial equation:

*ax*^3^ + *bx*^2^ + *cx*

Constants a,b,c,d refers to for 4th polynomial equation:

*ax*^4^ + *bx*^3^ + *cx*^2^ + *dx*

### Effect of aging

#### Anatomical Cross Sectional Area of the contractile part (ACSA)

##### Quadriceps

**Fig. 1:**
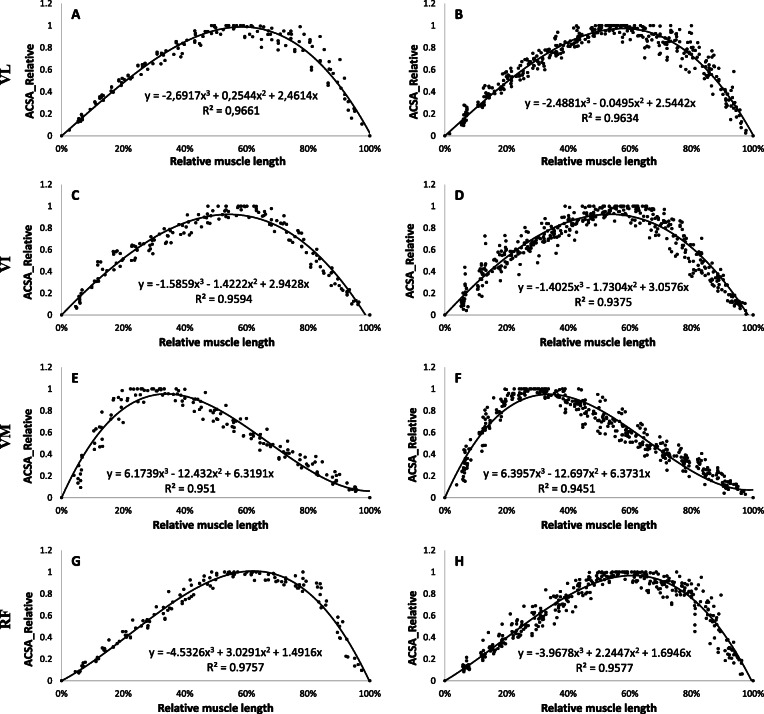
Regression equations obtained from the muscles of the quadriceps in young (**a, c, e, g**) and old (**b, d, f, h**) groups. Values are relative to maximum anatomical cross sectional area (ACSA) and muscle length. 0% of relative length corresponds to distal end (knee proximity) and 100% corresponds to proximal end (hip proximity). **a, b**: Vastus Lateralis (VL), **c, d**: Vastus Intermedius (VI), **e, f**: Vastus Medialis (VM) and **g, h**: Rectus Femoris (RF)

The evolution of the regression equations characterizing the mean evolution of the ACSA of the quadriceps muscles for young and old groups are presented in Fig. 1.

**Fig. 2:**
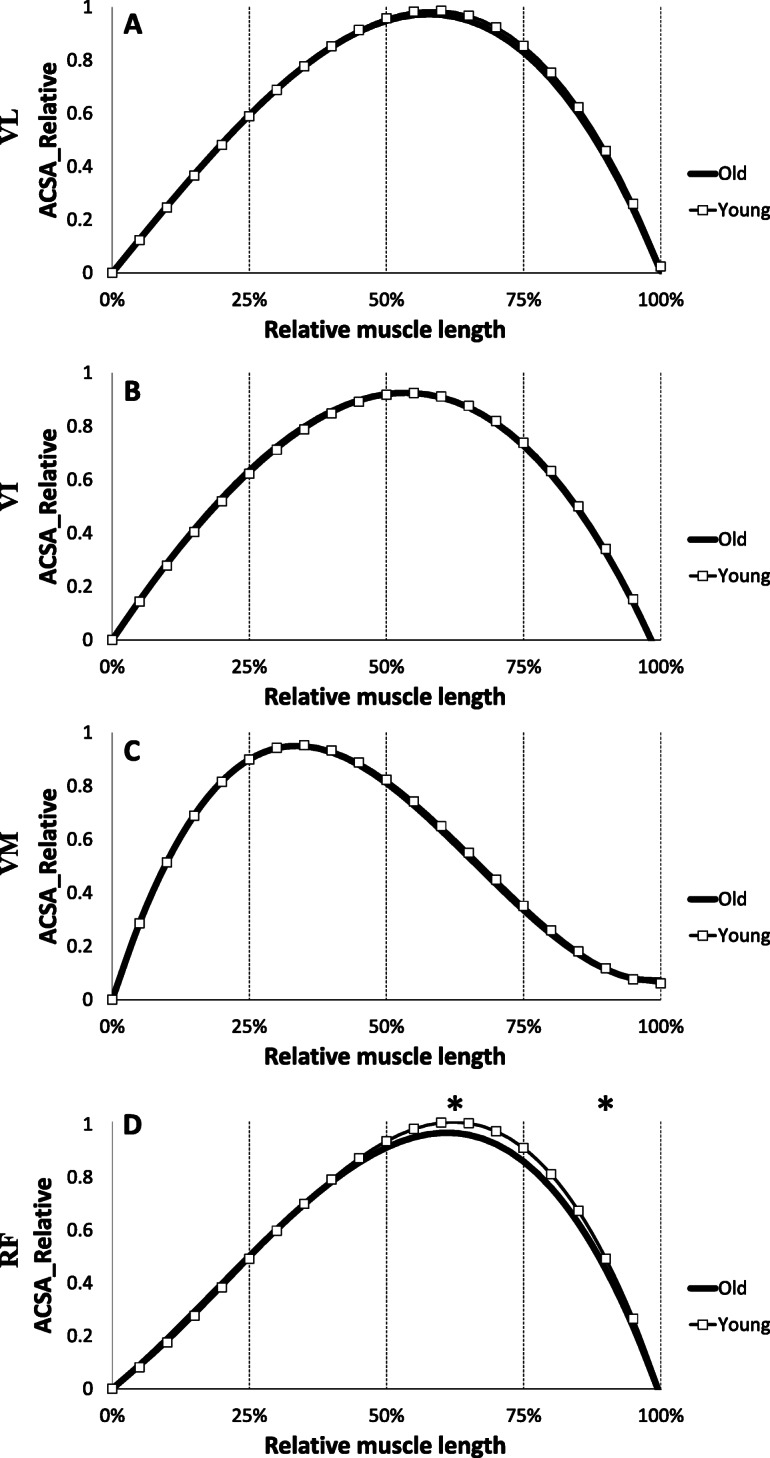
Comparison of regression equations obtained from the muscles of the quadriceps in young (grey) and old (black) groups. Values are relative to maximum anatomical cross sectional area (ACSA) and muscle length. A: Vastus Lateralis (VL), B: Vastus Intermedius (VI), C: Vastus Medialis (VM) and D: Rectus Femoris (RF); * Significant difference between young and old (*p* < 0.05)

Statistical analysis of the mean ACSA values calculated every 25% of the relative length of the VL, VI, and VM muscles shows no significant difference between the 2 groups (*p* > 0.05) (Fig. 2). Only RF muscle shows a significantly higher relative ACSA in the young than the elderly on the portion 50–75% and 75–100% from its relative length (*p* < 0.05).

##### Triceps surae

**Fig. 3:**
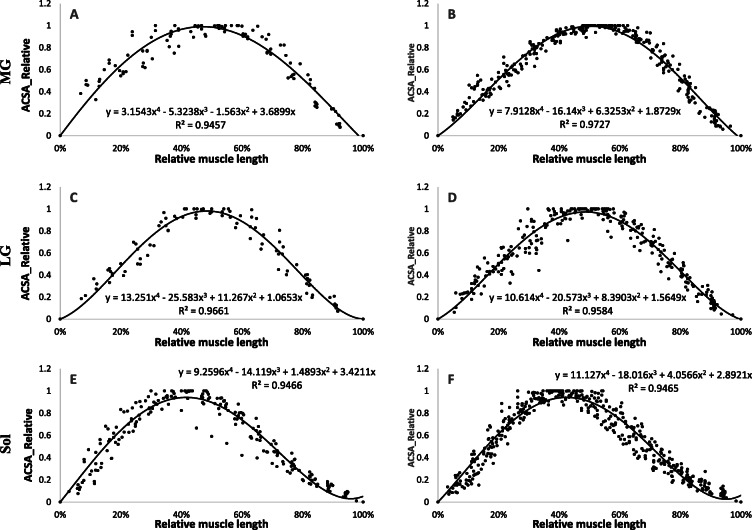
Regression equations obtained from the muscles of the triceps surae in young (**a, c, e**) and old (**b, d, f**) groups. Values are relative to maximum anatomical cross sectional area (ACSA) and muscle length. 0% of relative length corresponds to proximal end (knee proximity) and 100% corresponds to distal end (ankle proximity). **a,b**: Medial gastrocnemius (MG), **c,d**: Lateral gastrocnemius (LG) and **e,f**: Soleus (Sol)

**Fig. 4:**
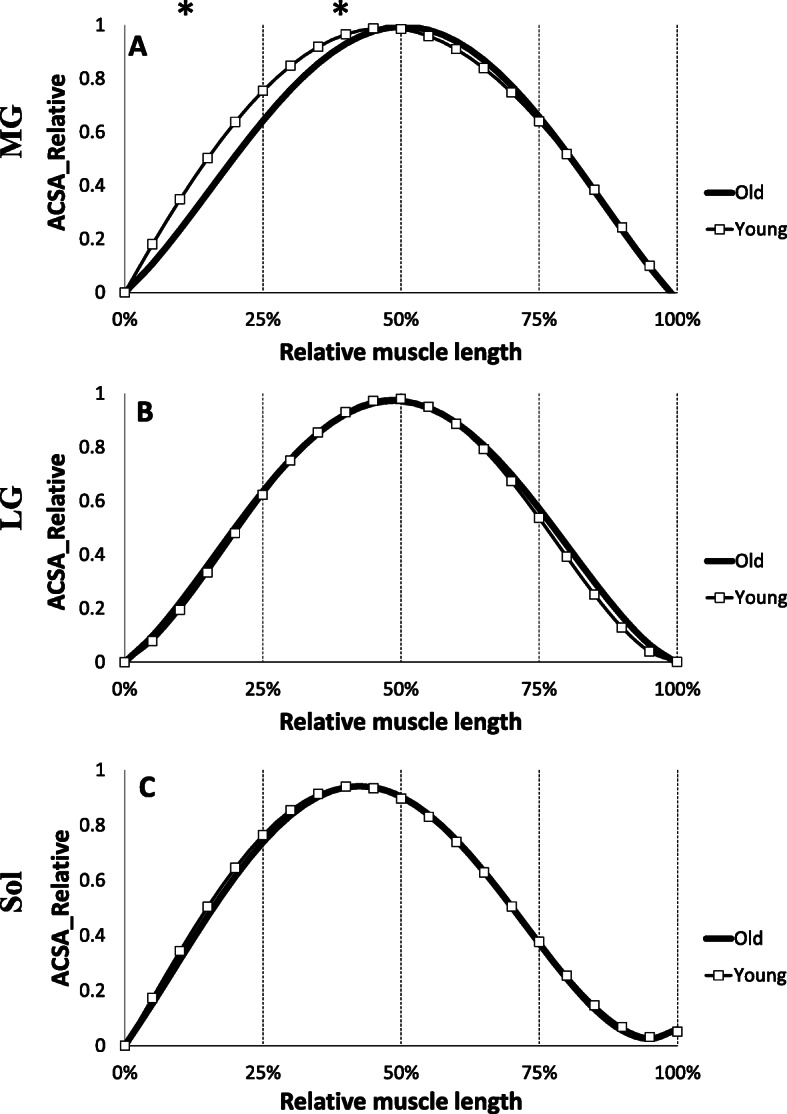
Comparison of Regression equations obtained from the muscles of the triceps surae in young (grey) and old (black) groups. Values are relative to maximum anatomical cross sectional area (ACSA) and muscle length. **a**: Medial gastrocnemius (MG), **b**: Lateral gastrocnemius (LG), **c**: Soleus (Sol); * Significant difference between young and old (*p* < 0.05)

Figure 3 shows the regression equations obtained from the three individual muscles composing the triceps surae. No significant difference in ACSA was observed over the entire length of the muscle between the young and elderly groups in LG and soleus muscles (Fig. 4). However, the mean relative ACSA values of MG muscle on the portion 0–25% and 25–50% of the relative muscle length were significantly higher for the young compared to the old group (*p* < 0.05).

#### Muscle volume

Muscle volumes presented here are based only on contractile material as determined and detailed previously.

##### Quadriceps

**Fig. 5:**
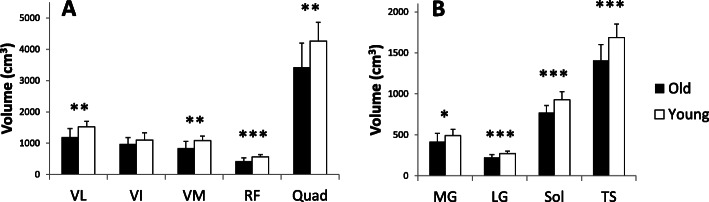
Quadriceps (**a**) and triceps surae (**b**) muscles volumes between old and young groups. Vastus Lateralis (VL), Vastus Intermedius (VI), Vastus Medialis (VM), Rectus Femoris (RF), Quadriceps (Quad), Medial gastrocnemius (MG), Lateral gastrocnemius (LG), Soleus (Sol), Triceps Surae (TS); * Significant difference between young and old (*p* < 0.05), ** (*p* < 0.01) and *** (*p* < 0.001)

For quadriceps muscles, the young group showed higher muscle volume in VL, VM and RF (+ 27.3%, + 28.1% (*p* < 0.01) and + 34.3% *p* < 0.001, respectively) compared to the old group (Fig. 5a). However, no difference in muscle volume between young and old could be identified for the VI (*p* > 0.05). Concerning the total volume of the quadriceps, the young group shows significantly higher values (+ 24.4%, *p* < 0.01) compared to the old group.

##### Triceps surae

Data in Fig. 5b shows a higher muscle volume in the young group for MG (+ 18.0%; *p* < 0.01), LG (+ 20.9%; *p* < 0.001) and Soleus (Sol) (+ 20.7%; *p* < 0.001). The total volume of triceps surae (TS) is also significantly higher in young participants than seniors (+ 19.9%; *p* < 0.01).

#### Non contractile tissue material

Table 5 shows the volume of fat and connective tissue in each muscle from both groups (young and old). The old group presents a higher absolute volume of non-contractile material than young men in the RF muscle (*p* < 0.05) and also in all TS muscles (MG: + 121% (*p* < 0.05); LG: + 87% (*p* < 0.01); Sol: + 87% (*p* < 0.01)), as well as a higher total volume of non-contractile material in TS (+ 96%, *p* < 0.01). However, no significant differences in non-contractile material volume between young and old could be identified for the VL, VI, VM and the total Quadriceps (*p* > 0.05). Moreover, a significant difference is observed between the relative volume of non-contractile material in relation to the total contractile volume on both muscle groups (quadriceps and TS) on old group (*p* < 0.05).

**Table 5:** Contractile part and connective tissue volume for each muscle in young and old group

Volume (cm^3^)	Young			Old		
	Contractile part	Fat and connective tissue	% fat / total contractile volume	Contractile part	Fat and connective tissue	% fat / total contractile volume
VL	1521.1 ± 272.5	57.9 ± 27.9	3.60%	1195.2 ± 178.7 **	56.5 ± 26.5	4.51%
VI	1099.6 ± 210.2	44.8 ± 25.1	3.86%	969.1 ± 230.2	44.5 ± 19.8	4.39%
VM	1078.4 ± 215.1	42.1 ± 20.9	3.70%	841.9 ± 147.9 **	39.4 ± 17.6	4.47%
RF	564.6 ± 113.0	21.6 ± 9.4	3.64%	420.4 ± 71.4 ***	67.5 ± 50.9 *	14.86%
**Total Quad**	**4263.8 ± 770.2**	**166.5 ± 82.5**	**3.70%**	3426.6 ± 598.6 **	**177.2 ± 71.7**	**4.92%**
MG	488.9 ± 101.6	17.7 ± 9.4	3.44%	414.5 ± 78.8 *	39.3 ± 32.1 *	8.66%
LG	271.1 ± 33.7	9.5 ± 5.1	3.31%	224.3 ± 29.2 ***	17.2 ± 7.1 **	7.14%
Sol	927.5 ± 90.1	31.5 ± 15.5	3.21%	768.4 ± 97.6 ***	58.9 ± 24.1 **	7.12%
**Total TS**	**1687.6 ± 192.6**	**58.7 ± 29.8**	**3.29%**	1407.3 ± 163.9 ***	115.4 ± 56.2 **	**7.58%** ^**#**^

*Significant difference between young and old (*p* < 0.05), ** (*p* < 0.01) and *** (*p* < 0.001)

# Significant difference between Quadriceps and Triceps Surae (TS) muscles group (*p* < 0.05). Data are presented as means ± SD

### Effect of training

#### Anatomical Cross Sectional Area of the contractile part (ACSA)

##### Quadriceps

**Fig. 6:**
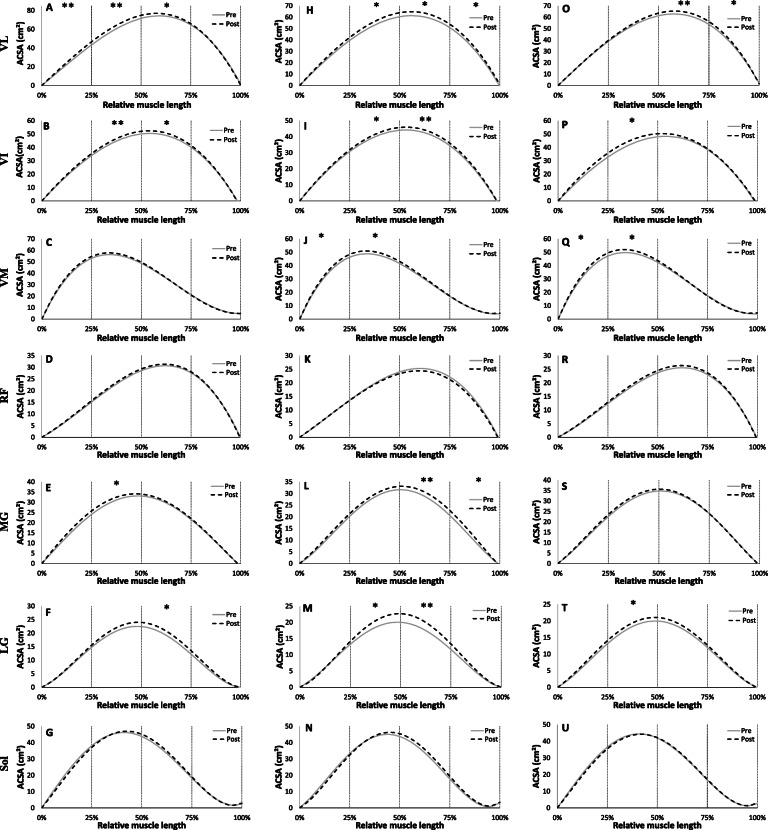
Evolutions of mean anatomical cross sectional areas (ACSA) values relative muscle length for each muscle of quadriceps and triceps surae groups for each training group between pre (grey line) and post training (black dashed line). **a,h,o**: VL of Y55,O55 and O80 respectively; **b,i,p**: VI; **c,j,q**: VM; **d,k,r**: RF; **e,l,s**: MG; **f,m,t**: LG; **g,n,u**: Sol. Vastus Lateralis (VL), Vastus Intermedius (VI), Vastus Medialis (VM), Rectus Femoris (RF), Medial gastrocnemius (MG), Lateral gastrocnemius (LG), Soleus (Sol); * Significant difference between pre and post training (*p* < 0.05) and ** (*p* < 0.01)

The evolution of ACSA of each quadriceps muscle for each training group are presented in Fig. 6. For the young group who trained at 55% of 1RM (Y55), statistical analysis of the mean ACSA values calculated every 25% of the relative length, shows a significant increase between pre and post training in the VL muscle on the portions 0–25%, 25–50% (+ 7.6%; *p* < 0.01) and 50–75% (+ 3.1%; *p* < 0.05) from its relative length. The 12-week training period induced a significant increase on muscle VI on the portions 25–50% (+ 6.0%; *p* < 0.01) and 50–75% (+ 3.6%; *p* < 0.05) of its relative length.

Concerning the old group who trained at 55% of 1RM (O55), over the intervals corresponding to 25% to 100% of the relative length, the mean ACSA values of the VL muscle were significantly higher following training (+ 6.7%; *p* < 0.05). Similarly, on the VI muscle, the mean ACSA increased on the portions 25 to 50% (+ 3.6%; *p* < 0.05) and 50 to 75% (5.1%; *p* < 0.01) after the training period. The VM muscle showed a significant increase on the portion 0 to 50% (+ 4.4%; *p* < 0.05).

For the group that trained at 80% 1RM (O80), mean ACSA values of VL muscle over intervals corresponding to 50% to 75% (+ 5.2%; *p* < 0.01) and 75 to 100% (+ 4.0%; *p* < 0.05) of the relative muscle length were significantly higher after training. VI and VM muscles also increased in the portion 25 to 50% after 12 weeks of training (+ 5.4% and + 4.1% respectively *p* < 0.05). However, training had no effect on average ACSA values of RF muscle (*p* > 0.05) regardless of training group.

##### Triceps surae

On the triceps surae muscles, the young group increased mean ACSA of the MG and LG muscles on the portion 25–50% and 50–75% (+ 3.7% and + 8.0% respectively; *p* < 0.05) respectively (Fig. 6). For the O55 group, the training program induced an increase in MG and LG ACSA on portion 50 to 75% (+ 10.9% and + 14.1% respectively; *p* < 0.01) and also 25 to 50% for LG (+ 9.0%; *p* < 0.05) and on 75 to 100% for MG (+ 6.4%; *p* < 0.05). LG muscle showed an increase but only on the portion 25 to 50% (+ 9.5%; *p* < 0.05) of its relative length after 12 weeks of training for the old group who trained at 80% of 1RM (O80). However, training had no effect on average ACSA values of soleus muscle (*p* > 0.05) whatever the training group.

#### Muscle volume

##### Quadriceps

**Fig. 7:**
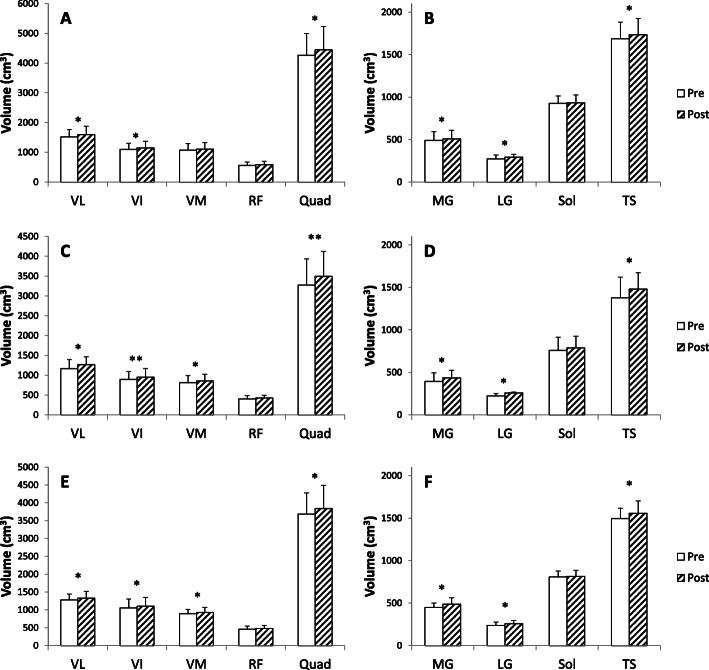
Pre and post muscles volumes for quadriceps (**a**: Y55, C: O55, **e**: O80) and triceps surae (**b**: Y55, **d**: O55, **f**: O80) on each training groups. Vastus Lateralis (VL), Vastus Intermedius (VI), Vastus Medialis (VM), Rectus Femoris (RF), Quadriceps (Quad), Medial gastrocnemius (MG), Lateral gastrocnemius (LG), Soleus (Sol), Triceps Surae (TS); * significant difference between pre and post training (*p* < 0.05), ** (*p* < 0.01) and *** (*p* < 0.001)

Figure 7 shows the variation of muscle volume before and after the training period on quadriceps for each training group. For the young group who trained at 55% of 1RM (Y55), the 12-week training period induced a significant increase in muscle volume on VL (+ 5.1%; *p* < 0.05), on VI (+ 4.8%; *p* < 0.05) and on the total quadriceps volume (+ 4.3%; *p* < 0.05). However, no difference in muscle volume was observed following training for VM (+ 3.1%; *p* = 0.08) and RF (+ 3.4%; *p* = 0.1). The results for the senior group who trained at 55% of 1RM (O55) show a significant increase in muscle volume on VL (+ 8.3%; *p* < 0.05), VI (+ 6.1%; *p* < 0.01), VM (+ 5.4%; *p* < 0.05), and total quadriceps (+ 6.7%; *p* < 0.01) following the training program. However, no difference was found after the training period for the RF in this training group (+ 5.6%; *p* = 0.12). For the old men group who trained at 80% (O80), training induced a significant increase in muscle volume on VL (+ 4.3%; *p* < 0.05), VI (+ 4.7%; *p* < 0.05), VM (+ 3.6%; *p* < 0.05), as well as on the total volume of the quadriceps (+ 4.2%; *p* < 0.05). However, training had no effect on the volume of RF (+ 3.8%; *p* = 0.2).

##### Triceps surae

Concerning the TS muscles (Fig. 7), the 12-week training period induced a significant increase in muscle volume on MG (+ 3.8%; *p* < 0.05), LG (+ 8.4%; *p* < 0.05) and on total TS volume (+ 2.8%; *p* < 0.05) for the young group who trained at 55% of 1RM (Y55). However, no difference in muscle volume was observed following training for soleus (*p* = 0.77). The senior group who trained at 55% of 1RM (O55) showed a significant increase in muscle volume on MG (+ 10.5%; *p* < 0.05), LG (+ 14.6%; *p* < 0.05) and total triceps surae volume (+ 7.5%; *p* < 0.05) following the training program. No difference was found after the training period for soleus muscle in this group (*p* = 0.47). The results of the O80 group showed a significant increase in muscle volume on MG (+ 8.2%, *p* < 0.05), LG (+ 9.0%; *p* < 0.05) as well as the total volume of TS (4.3%; *p* < 0.05) after the training period. In contrast, training had no effect on the volume of the soleus muscle (*p* = 0.58).

##### Comparison between gain in muscle volumes with training

The gains for each muscle on quadriceps and triceps surae between pre and post training are shown in Table 6. In the following A refers to differential effects of training with age and Qd refers to differential effects of training on each muscle on triceps surae compared to each muscle of Quadriceps. Gain in MG muscle volume was greater in old than in young men (A, *p* < 0.05) and likewise for gain in TS muscle in old group than in young men (A, *p* < 0.01 for O55 vs.Y55). A training effect on LG was observed (Qd, *p* < 0.05) with all groups increasing better than gains in volumes of each muscle of quadriceps group. Moreover, a multiple comparison procedure indicated a significantly greater increase in O55 than Y55 (*p* < 0.01) on average gains, while no difference between O55 and O80 was observed.

**Table 6:**
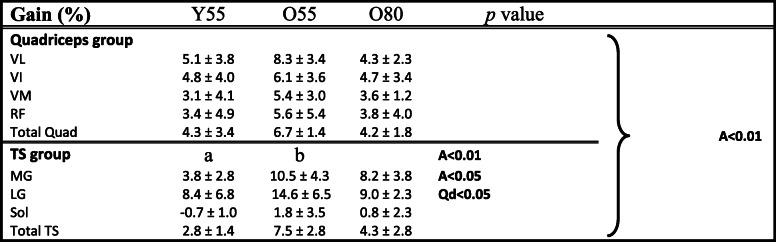
Gain (pre/post) in volume for each muscle for three training group

*p* values for effects are shown for two-way ANOVA (A: difference with age (O55 vs Y55), Qd: difference with each muscle of quadriceps group); Data are presented as means ± SD

## Discussion

The main objective of this study was to establish specific ACSA curves on young and old men to investigate whether age-related muscle loss in the lower limb muscles (quadriceps and TS) is a homogeneous phenomenon along muscle length or takes place in specific regions. The second objective was to determine whether a moderate-intensity strengthening exercise program (55% of 1RM) affects muscle adaptations in a population of young adults and healthy sedentary seniors; and to compare these effects to a high-intensity strengthening exercise program (80% of 1RM) in a healthy sedentary seniors population, having the same relative training volume as the moderate intensity training group. The few studies that have used a similar protocol [6, 7, 20] focused on only one muscle group, whereas the present study was able to compare for the first time the age effect as well as the impact of two strength training intensities on each individual muscle in two different muscle groups (quadriceps vs. TS).

### Effect of aging

A decrease in muscle mass occurs with age and a better understanding of this muscle atrophy is necessary to prevent or minimize this phenomenon. In this study, the distribution of ACSA normalized with the maximum ACSA was not affected by age on the VL, VI, and VM muscles. The results show that aging induces homogeneous effects throughout the excursion of these muscles. In contrast, we observed that the rectus femoris was affected by age at its proximal end (Fig. 2). One of the objectives of the study was to establish more precise ACSA evolution reference curves throughout the muscle excursion than those available in the literature for quadriceps muscle, and to establish reference curves for triceps surae. To our knowledge, only one study has reported equations of the muscle ACSAs [22], in which the authors presented third order polynomial equations only for quadriceps muscles. In addition, for the RF, due to limitations of the coil in this study, these authors had only part of the muscle excursion data, so the polynomial adjustments were applied without part of the real data. The polynomial adjustments reported in the present study are more precise because of a large sample (*n* = 27 for elderly), a finer segmentation (every 18.2 mm), and an exploration of the total length of each individual quadriceps muscle. We observe an atrophy of the RF at its proximal end with aging. While each of the four quadriceps heads is less voluminous with age, one study reported that the degree of atrophy of the RF was greater than that of the VI and VL in older women compared to younger women [37]. The RF is a bipennate and biarticular muscle, used during knee extension and hip flexion. It is not dominant in knee extension from a sitting position unlike the other three quadriceps leaders. In addition, the rectus femoris is a weaker hip flexor when the knee is elongated since it is already shortened. The different parts of the quadriceps have different functions and could therefore be subjected to different metabolic or mechanical stimuli due to muscle aging such as a decrease in the synthesis of contractile proteins and their greater degradation in fibres leading to a loss of actin-myosin filaments [1], higher atrophy of type II fibres [38] and a possible increase in density of type I fibres [39]. Concerning the quadriceps volume, we note a significant decrease of total volume with age as well as on the VL, VM and RF volumes (Fig. 5). Other authors have also shown a decrease in the volume of the four quadriceps muscles with age in men and women [37], or simply in the CSA of the quadriceps in men [40, 41], but also in the total thickness of the quadriceps [42]. This reduction in muscle volume is mainly due to a decrease in the penetration angle and length of the fascicles [11] as well as an atrophy of the CSA of muscle fibres [39] . Although some authors do not report changes in these same parameters by investigating only one quadriceps muscle (VL) [43], a reduction in pennation angle (26%) and in fascicles length (7%) with aging was observed in the VL [44]. This is in agreement with our results on TS, where the total volume was reduced accompanied by a decrease in the volume of the MG, LG and soleus with age. Other studies have reported similar decreases in the volume of TS with age [36, 45, 46] with the finding of changes in the fascicles length and pennation angle. When comparing the distribution of muscle ACSAs between the elderly and young groups, no difference was found in muscle shape for soleus and LG. However, age seems to have an effect on the evolution of CSA of the MG in its proximal part (Fig. 3). It has been reported that the pennation angle of the MG and the fascicles length are significantly decreased with age [11, 36]. In contrast, for soleus and LG muscles, only the pennation angle is reduced with age (Morse et al. 2005a). However, the mechanisms by which age modifies the ACSA curve on the proximal part of these muscles (RF and MG) remain unknown. Finally, regarding the evolution of the ACSA curves, our data underline the need to use different degree of regression curves for certain muscles to achieve the correct muscle shape (i.e. quadriceps or TS muscles), in order to more accurately detect effects of aging on muscle structure.

Moreover, in order to compare the fat infiltration with aging and avoid an overestimation of muscle volume when using these references curves, fat and connective tissue have been removed from all ACSAs and calculated as volume (Table **5**). Our results on fat and connective tissue content are in accordance with a previous study which only investigated the whole quadriceps muscle group between young and old men and women [34]. However, the volume of contractile part is clearly lower than our subjects (4-fold for elderly) and could explain the differences in percentage of non contractile/muscle volume observed for elderly in quadriceps muscle and for whole thigh in this study. The disparities in these findings might have been caused by differences in imaging modalities acquisition, or in ethnicities or lifestyles of the subjects as compared to Asian subject investigated in Yoshiko et al. [34]. Another study reported a non-contractile fraction in the TS of young men (4.2%) that was similar to the present data [47]. However these authors reported much greater increase in old men (24.2%) compared to our results. Another interesting result of our study was that the non-contractile content in the TS muscles was significantly greater than the quadriceps muscles (*p* < 0.05). Possible explanations for this discrepancy between TS and quadriceps muscles include muscle fiber types [48–50], architecture [24, 36, 51], or muscle contribution during daily activities [52]. The fat infiltration and connective tissue extracted from our MRI scans reflects extramyocellular lipid and this amount of lipid was 2 fold greater in plantar flexor muscle on young men than quadriceps muscle in adolescents [18, 47]. Thus, accumulation patterns of extramyocellular lipid may differ between quadriceps and triceps surae muscle in young and old adults and demonstrate significant age-related differences in non-contractile tissue for both muscles. Fat accumulation in muscle changes with age observed in this study is somewhat analogous to an increased visceral adiposity, which is related to an excessive production of pro-inflammatory cytokines that negatively affect muscle function [53]. However, it remains unknown whether muscular fat is simply a marker of metabolic dysfunction of adjacent skeletal muscle or whether this fat depot plays a more active role in sarcopenia or muscle contractility.

### Effect of training

Through the acquisition of MRI images of all muscles, we were able to follow accurately the adaptation of the CSA and volumes of the quadriceps and TS muscles on a group of young and seniors following a training period at different intensities (55% vs 80% of 1RM). To our knowledge, the present study is the first reporting changes in the ACSAs of the quadriceps and TS throughout their entire excursion after a training period. Changes in the ACSA of the quadriceps muscles with training indicate that the VL muscles of O55 and O80 groups have enlarged on the medial and proximal portion, while Y55 showed a hypertrophy of this same muscle on the medial and distal part. These results indicate that each of the investigated muscle can enlarge with training in different areas or a specific area. In the various studies reporting an increase in quadriceps CSA, this is generally obtained from a transverse section obtained using ultrasound or MRI techniques at 30% [6, 54] or 50% of the femur length [7, 55] from the patella; or 50% of the distance between the proximal and distal apexes of the myotendinous junction for CSA of GM and GL [11]. From data of the present study, we were able to highlight for the first time that a period of resistance training induces different adaptations of the proximal, mid-proximal, mid-distal and distal parts with age and training intensity. This also highlights the necessity to realize several cross-sections to observe finer adaptations depending on the muscle being investigated after training, rather than just one section as has often been the case in previous studies on the lower limb [6, 7, 54–56]. Our study shows that analyzing only one muscle slide does not reflect the adaptation of the whole muscle and then cannot be used the follow muscle adaptations. Based on our results, a good practice would be to get at least one muscle slide in each of the main parts of the investigated muscle. In our study, the total volume of the quadriceps is significantly increased with training for each training group (+ 4.3% for Y55; + 6.7% for O55; + 4.2% for O80). We also observed an increase in volume for each of the four muscles, but significantly different only on VL and VI for the Y55 group, and on VL, VI, and VM for the O80 and O55 groups. Regarding the young participants, several studies have shown an increase in quadriceps muscle volume at high intensity (≥80% of 1RM) after resistance training from 9 to 16 weeks (+ 10.2% for Blazevich et al. [26]; + 5.6% for Erskine et al. [12]; 7.2% for Bellamy et al. [57]). In their study, Blazevich et al. [26] also reported an increase in the volume of VL and VM of 11% and 15% respectively following training. Moreover, 24-weeks of resistance training at 5RM also induced an increase in the whole volume of the thigh muscles (quadriceps + hamstrings) by 4.1% in young men with an increase in the volume of VM by 2% [7]. In our study, we showed that a 12 week of resistance training at 55% of 1RM induced a 4.3% increase in the quadriceps muscle volume in young men. Although our training period lasted 12 weeks, which is twice less than the experimental protocol as compared to Roth et al. (2001), the quadriceps muscle volume gain in our study is equal to that reported by these authors for a training period twice as long. As mentioned by other authors, the increase in muscle volume is not proportional to the duration of training. After 3 months of training, the increase in muscle volume becomes low or even non-existent [58]. More recently, a meta-analysis has been conducted to compare changes in strength and hypertrophy between low (≤60% of 1RM) vs. high-load (> 60% of 1RM) resistance training protocols [59]. These authors concluded that changes in muscle hypertrophy due to strength training were similar between conditions (low vs high-load). The findings indicate that maximal strength benefits are obtained from the use of heavy loads while muscle hypertrophy can be equally achieved across a spectrum of loading ranges. However, a limitation of this meta-analysis is that the authors included studies with men and women, young and old and trained/untrained participants and pooled together all these studies without separately investigating the effect of these parameters following to training. In addition, interestingly our study shows for the first time in elderly people that high intensity training (80% of 1RM) does not bring a greater increase in muscle volume compared to the group of seniors who trained at moderate intensity (55% of 1RM). Our results obtained for the moderate intensity training group are in accordance with Van Roie et al. [60] that showed an increase (+ 2.6%) in muscle volume of the thigh (quadriceps + hamstrings) for a mixed group of seniors following a 12-weeks training period at a low intensity (~ 30% of 1 RM). Concerning the total volume of the triceps surae, both groups of seniors (O55 and O80) show an increased in TS muscle volume (+ 4.3% for O80; + 7.5% for O55). The volume of LG and MG was higher for the three training groups with always a greater hypertrophy on LG as compare to MG and SOL, or the four individual muscles of the quadriceps. However, we also reported that the MG in the two old groups shows greater hypertrophy than the young group (A < 0.05). Indeed, we observed a significant difference with age on volume gains on TS muscle group between young and old training group (*p* < 0.01 for O55 vs. Y55), without differences between O80 vs O55.

Morse et al. [20] evaluated the effect of long-term training (52 weeks) at an intensity of 8RM on a population of male seniors and showed an increase in total TS volume of + 15%, with an increase in LG, MG, and soleus volume of + 23, + 19, and + 10.8% respectively. Our study also showed a significant muscle adaptation in term of muscle volume for TS and quadriceps muscles following a medium-term training period (12 weeks). These results indicate that, despite aging, TS muscles are capable of hypertrophy following the application of a repeated mechanical stimulus. It is very interesting to note that, our study reveals that O80 group did not obtain a greater increase in all muscle volumes compared to the group of seniors who trained at moderate intensity (55% of 1RM) (Table 6). The main cause of this hypertrophy is likely an increase in the size of muscle fibres linked to changes in anabolic signaling and an increase in protein synthesis [10]. As previously mentioned, it has been shown that the rate of muscle protein synthesis reaches a maximum from 60% of 1RM in the elderly, unlike younger participants who see their rate continue to increase up to 90% of 1RM. The results obtained in the present study on the entire muscular system in vivo seem to be in agreement with Kumar et al. [10] results on the rate of protein synthesis. Thus, the present study does confirm that there would have in the elderly men a threshold in terms of stimulation intensity beyond which no additional gain is expected on muscle structure. This threshold would therefore be around 55 to 60% of 1RM in senior males.

There are some inherent limitations in this study design. Although the subjects recruited are sedentary, natural selection cannot be excluded such as the effects of environmental living conditions during growth and aging periods of the older compared to younger recruited subjects may have an influence on the present study findings. Another limitation of this study was that it remain difficult to clearly distinguish the VM and the VL muscles in some subjects on MRI images near proximal insertions, possibly due to fusion of these muscles, as seen in cadaveric specimens [61]. Although the use of regression equations to determine muscle volume can be considered as a limitation. Indeed, the errors have been minimized with the use of 3rd or 4th degree polynomial adjustment.

### Reliability

Despite the fact that we do not have an untrained control group for the 3 month training period, we justify this pragmatic choice by instead including two series of measurements with an interval of 1 month before training to avoid additional recruitment. Values of each parameter obtained during the two pre-training sessions did not differ. This demonstrates that repeated measurements under similar conditions generate similar results. Furthermore, the ICCs (> 0.79) as well as the RMS differences (< 1.0 cm^2^ ± 0.5) for the muscle ACSA can be achieved by averaging at least two trials. This indicates an acceptable precision of the used methodology. Moreover, the accuracy of using serial ACSA scans from MRI for the measurement of muscle volume has been reported previously [19, 33].

## Conclusion

To our best knowledge, this study reports for the first time data on specific reference curves of ACSA in young and old men for the quadriceps and triceps surae muscles. These reference curves could then be used for estimating muscle volume by a single scan, with however the necessity to carry out several CSA if the objective is to follow the evolution of participants following an intervention period. We highlight that muscle loss with aging is region-specific for some muscles and is a homogeneous phenomenon for others. We also reported that there was no difference for muscle volume gains between moderate or high intensities with equivalent strength training volume for elderly men. Also, these two training intensities are more effective in terms of gain on TS muscle than quadriceps.

The present results suggest that physical exercise at moderate intensity (55 to 60% of 1RM), can be enough to counteract the aging related loss of muscle mass, with higher training intensity not leading to additional muscle gains. This hypothesis warrants further study in order to maintain a quality of life for the elderly person.

## Data Availability

The datasets used and/or analysed during the current study are available from the corresponding author (adrien.letocart@yahoo.fr) on reasonable request.

## Abbreviations

1RM: One Repetition Maximum
ACSA: Anatomical cross-sectional area
ANOVA: Analysis of variance
BMI: Body mass index
CSA: Cross-sectional area
ICC: Intraclass correlation
LG: Lateral gastrocnemius
MG: Medial gastrocnemius
MRI: Magnetic resonance imaging
O55: Old men who trained at 55% of 1RM
O80: Old men who trained at 80% of 1RM
RF: Rectus Femoris
RMS: Root mean square differences
ROI: Region of interest
Sol: Soleus
TS: Triceps surae
VI: Vastus Intermedius
VL: Vastus Lateralis
VM: Vastus Medialis
Y55: Young men who trained at 55% of 1RM
